# Noninvasive 3-Dimensional ^1^H-Magnetic Resonance Spectroscopic Imaging of Human Brain Glucose and Neurotransmitter Metabolism Using Deuterium Labeling at 3T: Feasibility and Interscanner Reproducibility

**DOI:** 10.1097/RLI.0000000000000953

**Published:** 2023-02-04

**Authors:** Fabian Niess, Lukas Hingerl, Bernhard Strasser, Petr Bednarik, Dario Goranovic, Eva Niess, Gilbert Hangel, Martin Krššák, Benjamin Spurny-Dworak, Thomas Scherer, Rupert Lanzenberger, Wolfgang Bogner

**Affiliations:** From the ∗ High Field MR Center, Department of Biomedical Imaging and Image-Guided Therapy, Medical University of Vienna, Vienna, Austria; † Danish Research Centre for Magnetic Resonance, Centre for Functional and Diagnostic Imaging and Research, University Hospital Amager and Hvidovre, Hvidovre, Denmark; ‡ Department of Radiology, Centre for Functional and Diagnostic Imaging and Research, Copenhagen University Hospital Amager and Hvidovre, Hvidovre, Denmark; § Department of Neurosurgery; ∥ Department of Medicine III, Division of Endocrinology and Metabolism; ¶ Department of Psychiatry and Psychotherapy, Comprehensive Center for Clinical Neurosciences and Mental Health (C3NMH), Medical University of Vienna, Vienna, Austria.

**Keywords:** magnetic resonance spectroscopic imaging, deuterium labeling, quantitative exchange label turnover, downstream glucose metabolism, neurotransmitter, brain, glutamate, glutamine, deuterium metabolic imaging

## Abstract

Noninvasive, affordable, and reliable mapping of brain glucose metabolism is of critical interest for clinical research and routine application as metabolic impairment is linked to numerous pathologies, for example, cancer, dementia, and depression. A novel approach to map glucose metabolism noninvasively in the human brain has been presented recently on ultrahigh-field magnetic resonance (MR) scanners (≥7T) using indirect detection of deuterium-labeled glucose and downstream metabolites such as glutamate, glutamine, and lactate. The aim of this study was to demonstrate the feasibility to noninvasively detect deuterium-labeled downstream glucose metabolites indirectly in the human brain via 3-dimensional (3D) proton (^1^H) MR spectroscopic imaging on a clinical 3T MR scanner without additional hardware.

**Materials and Methods:**

This prospective, institutional review board–approved study was performed in 7 healthy volunteers (mean age, 31 ± 4 years, 5 men/2 women) after obtaining written informed consent. After overnight fasting and oral deuterium-labeled glucose administration, 3D metabolic maps were acquired every ∼4 minutes with ∼0.24 mL isotropic spatial resolution using real-time motion-, shim-, and frequency-corrected echo-less 3D ^1^H-MR spectroscopic Imaging on a clinical routine 3T MR system. To test the interscanner reproducibility of the method, subjects were remeasured on a similar 3T MR system. Time courses were analyzed using linear regression and nonparametric statistical tests. Deuterium-labeled glucose and downstream metabolites were detected indirectly via their respective signal decrease in dynamic ^1^H MR spectra due to exchange of labeled and unlabeled molecules.

**Results:**

Sixty-five minutes after deuterium-labeled glucose administration, glutamate + glutamine (Glx) signal intensities decreased in gray/white matter (GM/WM) by −1.63 ± 0.3/−1.0 ± 0.3 mM (−13% ± 3%, *P* = 0.02/−11% ± 3%, *P* = 0.02), respectively. A moderate to strong negative correlation between Glx and time was observed in GM/WM (*r* = −0.64, *P* < 0.001/*r* = −0.54, *P* < 0.001), with 60% ± 18% (*P* = 0.02) steeper slopes in GM versus WM, indicating faster metabolic activity. Other nonlabeled metabolites showed no significant changes. Excellent intrasubject repeatability was observed across scanners for static results at the beginning of the measurement (coefficient of variation 4% ± 4%), whereas differences were observed in individual Glx dynamics, presumably owing to physiological variation of glucose metabolism.

**Conclusion:**

Our approach translates deuterium metabolic imaging to widely available clinical routine MR scanners without specialized hardware, offering a safe, affordable, and versatile (other substances than glucose can be labeled) approach for noninvasive imaging of glucose and neurotransmitter metabolism in the human brain.

Impairment of glucose (Glc) metabolism in the human brain, that is, a shift from aerobic Glc utilization toward anaerobic pathways (Warburg effect), has been linked to several pathologic conditions observed in, for example, ischemia and tumors.^1^ Anaerobic glycolysis plays an important role during early stages of dementia^2^ and neuropsychiatric disorders such as schizophrenia and depression.^3^

[^18^F]-Fluorodeoxyglucose (FDG) positron emission tomography (PET) is the current gold standard in clinical routine for assessing tissue-specific Glc uptake, but it requires invasive administration of unstable radioactive tracers and does not provide information on the dynamics of Glc downstream metabolites, for example, oxidative neurotransmitter synthesis of glutamate (Glu) and glutamine (Gln), or anaerobic lactate production, in, for example, tumors due to Glc trapping of [^18^F]FDG. Therefore, a fully noninvasive approach to reliably map the brain Glc metabolism is of critical interest for clinical research and routine application.^4^

Deuterium metabolic imaging (DMI)^5,6^ and quantitative exchanged label turnover (QELT)^7–10^ are novel magnetic resonance (MR) techniques to noninvasively image Glc metabolism in animals and the human brain using deuterium-labeled Glc as tracer, which are able to separate healthy oxidative from pathologic anaerobic pathways, by simultaneously detecting the respective metabolic products Glu + Gln and lactate.^4^ Deuterium metabolic imaging detects deuterium enrichment in the brain tissue directly via ^2^H-MR spectroscopy (MRS), whereas, indirectly, QELT detects a decrease in signal intensities in conventional ^1^H-MR spectra due to deuterium-to-proton exchange of labeled and unlabeled molecules, comparably as already performed using ^13^C-labeled Glc.^11^ Recently, DMI has been translated to clinical field strength of 3T^12^ with a nominal isotropic resolution of 33 mL and an acquisition time of 10 minutes for each 3-dimensional (3D) dataset. Quantitative exchanged label turnover features higher signal-to-noise ratio (SNR) and spatial resolution (0.12 mL) with shorter acquisition times (~3 minutes^7^) compared with DMI, while additionally detecting nonlabeled metabolites, but so far has been applied in humans only at ultrahigh field strength of ≥7T.^7,8^

Translating this QELT method to widely available clinical MR scanners (≤3T) without the need for additional expensive hardware would offer radiologists immediate access to a fully noninvasive and affordable approach to map Glc downstream metabolism.

This study demonstrates the feasibility to noninvasively image Glc downstream metabolism in the human brain on a clinical 3T MR scanner using the QELT approach with time-resolved 3D proton (^1^H) MR spectroscopic imaging (MRSI) and deuterium-labeled Glc.

## METHODS

### Participants

This study was approved by the ethic commission of the Medical University of Vienna and written informed consent was obtained from all participants. Seven healthy participants (mean age, 31 ± 4 years; 5 men; body mass index, 22 ± 1 kg/m^2^) were recruited consecutively at the Department of Biomedical Imaging and Image-Guided Therapy of the Medical University Vienna with the following inclusion criteria: no contraindication to 3T MR imaging (MRI), history of neurological or psychiatric disorders, claustrophobia, metabolic disorders, or metal implants.

### Study Protocol

The study included an MRI protocol and blood Glc sampling. Magnetic resonance imaging was performed in the morning after overnight fasting and immediately after oral tracer administration using deuterium-labeled Glc ([6,6′]-^2^H-Glc; 0.8 g/kg body weight in 200 mL water). Six subjects were remeasured within 6 to 9 months to assess the repeatability of the method. All subjects consumed the labeled Glc under 1 minute shortly before being moved into the scanner. Capillary puncture blood sampling from the toe (ie, most accessible sampling site in the MR scanner) was performed 6 times over the course of ~90 minutes (we aimed for standardized time points of 0, 15, 30, 45, 60, and 100 minutes) using 2 standard strip glucometers (Verio, OneTouch) for cross-checking.

### MRI Protocol

All measurements were conducted on clinical routine 3T MR systems (Prisma-FIT and Prisma for test and retest measurements, respectively) using a 64-channel receive head coil (Siemens Healthineers, Erlangen). Preparation scans included an automated alignment localizer followed by echo planar imaging reference scans to set up the volumetric navigator sequence used for real-time motion correction.^13^ A previously developed 3D echoless (free induction decay [FID]) MRSI sequence with automatic interleaved real-time motion-, shim-, and frequency drift-correction and fast concentric ring trajectory readout^14,15^ was used to acquire 14 consecutive 3D datasets over the course of ~60 minutes: 0.8 milliseconds acquisition delay, 950 milliseconds repetition time, 0.24 mL isotropic nominal voxel volume (matrix size, 32 × 32 × 21; field of view, 200 × 200 × 130 mm^3^; volume of interest, 200 × 200 × 55 mm^3^ centered around the posterior cingulate region), and 4 minutes 14 seconds acquisition time per repetition (for details, see table, Supplemental Digital Content 1, http://links.lww.com/RLI/A798^16^). Water suppression was performed using conventional water suppression Enhanced through T1 effects scheme,^17^ whereas unsuppressed water reference signals (20 FID points) were acquired during each T_R_ for determination of coil sensitivities.

After the MRSI scan, a 3D T1-weighted magnetization-prepared rapid gradient echo readout scan was performed: 1800 milliseconds repetition time, 2.27 milliseconds echo time, 900 milliseconds inversion time, 1 mm^3^ isotropic nominal voxel volume (field of view, 250 × 250 × 350 mm; matrix size, 256 × 256 × 208), 3-fold parallel acquisition acceleration (Generalized Autocalibrating Partial Parallel Aquisition), and 2 minutes 38 seconds acquisition time.

### Data Reconstruction

An in-house developed software pipeline (MATLAB R2021, Python 3.10) was used for automatic data processing, including k-space in-plane convolutional gridding,^18^ noise decorrelation, channel-wise lipid decontamination,^19^ coil combination,^20^ and spectral fitting (4.2–1.8 ppm) using LCModel (version 6.3). Voxel-wise spectral fitting was performed for all 3D datasets and time points, without the use of spectral subtraction. Quantification results of voxels that did not fulfill the quality control criteria, that is, full width at half maximum <0.1 ppm, signal-to-noise ratio >15, and Cramer-Rao Lower Bounds (CRLBs) <20%, were excluded from the analysis.

### Metabolite Quantification

A modified basis set was used featuring 17 typical metabolites (ie, creatine, glycerophosphocholine, glutathione, myo-inositol, *N*-acetylaspartate, *N*-acetylaspartylglutamate, phosphocholine, phosphocreatine, taurine, aspartate, γ-aminobutyric acid, Glc*α*, Glc*β* Glu, Gln, and lactate) of neurochemical profile (including measured macromolecular contributions),^21^ but this work focuses on few selected metabolites of interest: Glu + Gln (Glx), total creatine (tCr), and total *N*-acetylaspartate (tNAA). During metabolic utilization of deuterium-labeled Glc ([6,6′]-^2^H-Glc), downstream metabolites incorporate deuterium at specific carbon positions. In case of oxidatively synthesized Glu and Gln, deuterium labeling occurs only at the fourth carbon position (Glu_4_: 2.34 ppm, Gln_4_: 2.44 ppm), eventually leading to a signal intensity decrease of the respective resonances, whereas resonances associated with protons at the second and third position (Glu_2_: 3.75 ppm, Glu_3_: 2.10 ppm, Gln_2_: 3.77 ppm, and Gln_3_: 2.13 ppm) remain stable.

Therefore, resonances representing Glu and Gln in the basis set were separated into Glu_4_ and Glu_2 + 3_ and Gln_4_ and Gln_2 + 3_, respectively, and then summed to Glx_4_ and Glx_2 + 3_, representing labeled and unlabeled resonances, respectively. Separation was performed by simulating Glu and Gln with fully deuterated Glu_4_ and Gln_4_ (equals Glu_2 + 3_ and Gln_2 + 3_: both protons at the fourth carbon position are replaced with deuterons) and subtracting it from regular Glu and Gln to obtain Glu_4_ and Gln_4_. Therefore, a linear combination equals regular Glu and Gln molecules taking J-coupling effects into account. Similarly, only the sixth carbon position of Glc is labeled, and therefore, Glc_6_ (labeled) and Glc_1–5_ (unlabeled) components were introduced. Labeling of Glc on other carbon positions than [6,6] is lost during metabolic utilization and is not detectable. Metabolic maps for Glx_4_ were calculated as a voxel wise ratio reference to tCr. Metabolite concentration estimates of regionally averaged Glx_4_ for gray (GM) and white (WM) matter are given in absolute units (mM), referenced to assumed concentrations of tCr (GM: 7.5 mM, WM: 5.7 mM) and relaxation times from literature (T_1_: tCr_GM/WM_ = 1.46/1.24 seconds, Glx_GM/WM_ = 1.27/1.24 seconds, T_2_: tCr_GM/WM_ = 201 milliseconds/198 milliseconds, Glx_GM/WM_ = 134 milliseconds/148 milliseconds).^22–24^ Glx_2 + 3_, tCr, and tNAA concentrations are given in arbitrary units.

### Time Course Analysis

Three-dimensional metabolic maps were created for all 14 time points representing the metabolic dynamics over ~60 minutes with a high time resolution of ~4 minutes. Maps were coregistered to anatomical T1-weighted images. Regional segmentation for GM and WM was automatically performed using the Functional Magnetic Resonance Imaging of the Brain Automated Segmentation Tool algorithm^25^ on T1-weighted 3D images and down-sampled to 32 × 32 using MINC tools (MINC tools, v2.0, McConnell Brain Imaging Center, Montreal, QC, Canada). Partial volume effects were minimized using a threshold of 80% for GM and WM voxels, respectively.

Temporal metabolite signal evolution was investigated voxel-wise and over the whole GM or WM in each participant, given as mean ± standard deviation. To estimate temporal stability of metabolite concentration fits, coefficients of variation were calculated over the entire MRSI scan (60 minutes and 14 time points) for stable nonlabeled metabolites (Glx_2 + 3_, tCr, tNAA).

### Statistical Analysis

Linear regression analysis was performed between time and quantified metabolite signals, for example, deuterium-labeled resonances (Glx_4_). Differences between the first and last time points and between GM and WM groups were evaluated using Wilcoxon signed-rank test and Mann-Whitney *U* test between segmented GM and WM voxels. The statistical significance threshold was *P* < 0.05. Linear fitting and statistical tests were performed using Python 3.10 (www.python.org, packages: scipy.stats).

## RESULTS

### Study Protocol

All preparation scans were completed 6 ± 2 minutes after tracer administration. The subsequent MRSI sequence acquired 3D datasets consecutively every ~4 minutes without interruption or operator interaction. Capillary blood sampling and analysis were performed in 5 participants.

### Time Course Analysis

**FIGURE 1:**
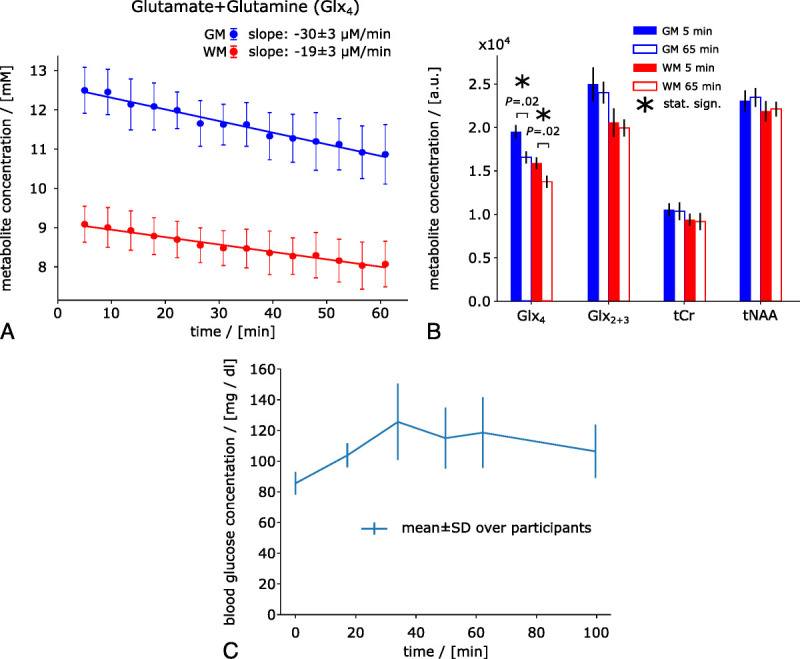
Time courses of deuterium-labeled Glx (Glx_4_) signal concentrations in mM (from whole GM and WM averaged over all participants). A decrease in Glx_4_ over time can be observed in both GM and WM. The decrease in Glx_4_ was 60% ± 18% faster in GM compared with WM (A). First and last measurement (first at ~5 minutes, last at ~65 minutes) of deuterium-labeled (Glx_4_) and unlabeled (Glx_2 + 3_, tCr, tNAA) resonances shown as regional means over whole GM and WM and over all participants (B). Averaged time course of blood Glc concentration over all participants increasing from 86 ± 7 mg/dL to 126 ± 25 mg/dL at 34 minutes (averaged sampling time points) after tracer administration before gradually decreasing toward baseline (C).

Moderate to strong negative correlation with time was observed for Glx_4_ in GM (*r* = −0.64, *P* < 0.001) and WM (*r* = −0.54, *P* < 0.001), with 60% ± 18% steeper slopes in GM compared with WM (*P* = 0.02), representing faster signal decay (Fig. 1a). Individual results of linear regression analysis are listed for test and retest measurements (see Table 1).

**TABLE 1:** Initial Concentrations and Linear Regression Results per Participant and Region for Test and Retest Measurement Bouts

Metabolite	Initial Concentrations, mM	Slopes, μM/min	Correlation Coefficient, *r*	*P*
Glx4				
Test				
Participant 1	11.5/8.6	−31.6/−23.3	−0.99/−0.98	<0.001/<0.001
Participant 2	13.3/9.6	−31.2/−20.4	−0.96/−0.97	<0.001/<0.001
Participant 3	12.8/9.6	−24.0/−15.1	−0.89/−0.86	<0.001/<0.001
Participant 4	13.0/9.5	−27.7/−17.3	−0.94/−0.87	<0.001/<0.001
Participant 5	11.9/8.4	−30.8/−21.9	−0.95/−0.95	<0.001/<0.001
Participant 6	12.3/8.9	−35.2/−19.8	−0.93/−0.96	<0.001/<0.001
Participant 7	12.7/9.0	−27.0/−14.1	−0.97/−0.92	<0.001/<0.001
Retest				
Participant 1	11.2/8.2	−13.5/−11.6	−0.64/−0.72	0.014/0.003
Participant 2	11.8/8.7	−25.2/−14.9	−0.93/−0.86	<0.001/<0.001
Participant 3	12.1/9.1	−33.8/−26.8	−0.88/−0.81	<0.001/<0.001
Participant 4	13.2/9.8	−37.0/−27.8	−0.96/−0.96	<0.001/<0.001
Participant 5	11.3/8.2	−22.6/−14.5	−0.87/−0.89	<0.001/<0.001
Participant 6	–	–	–	–
Participant 7	12.5/8.9	−20.2/−16.8	−0.85/−0.97	<0.001/<0.001

Data are from gray/white matter.

Glx indicates glutamate + glutamine.

Sixty-five minutes after tracer administration, Glx_4_ had decreased by −1.63 ± 0.3 mM (−13% ± 3%, *P* = 0.02) and −1.0 ± 0.3 mM (−11% ± 3%, *P* = 0.02) compared with the initial time point in GM and WM, respectively, whereas no changes were found for Glx_2 + 3_, tCr, or tNAA (GM/WM: Glx_2 + 3_, *P* = 0.08/0.16; tCr, *P* = 0.47/0.30; tNAA, *P* = 0.16/0.81) (Fig. 1b).

Blood Glc concentration increased from 86 ± 7 mg/dL at baseline to 126 ± 25 mg/dL (*P* = 0.068) at 34 minutes (averaged sample time points) after tracer administration, followed by a gradual decrease toward baseline (Fig. 1c).

Nonlabeled metabolite resonances, for example, Glx_2 + 3_, tCr, and tNAA, featured high temporal stability with coefficients of variation <2% (Glx_2 + 3_, 1.9% ± 0.6%; tCr, 1.5% ± 0.8%; and tNAA, 1.1% ± 1%).

Comparison between results from test and retest experiments including a Bland-Altman comparison for absolute Glx_4_ in mM is shown in Supplementary Digital Content, Figures 1 and 2, http://links.lww.com/RLI/A798 and http://links.lww.com/RLI/A798, respectively.

**FIGURE 2:**
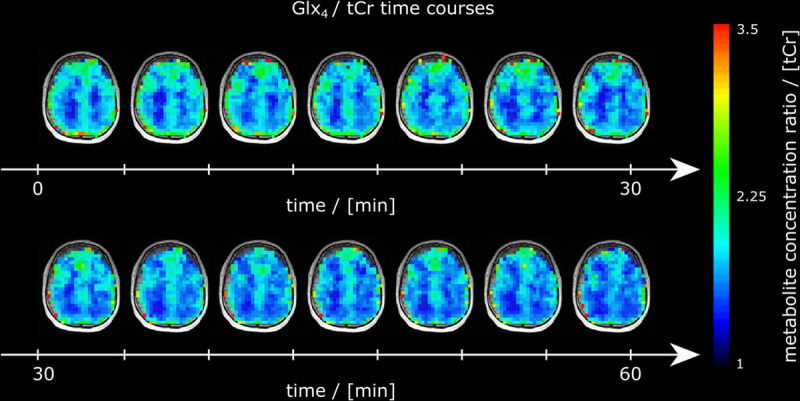
Time courses of axial Glx ratio maps referenced to tCr (Glx_4_/tCr) maps from 1 representative subject over the entire measurement visualizing the image intensity decrease over time due to deuterium labeling.

Representative axial Glx_4_/tCr ratio maps are shown for all time points from 1 representative subject (Fig. 2). A continuous signal intensity decrease over time due to deuterium labeling was visually discernable. Metabolic map time courses (Glx_4_/tCr ratio maps) from all participants are shown in Supplemental Digital Content Figure 3, http://links.lww.com/RLI/A798.

**FIGURE 3:**
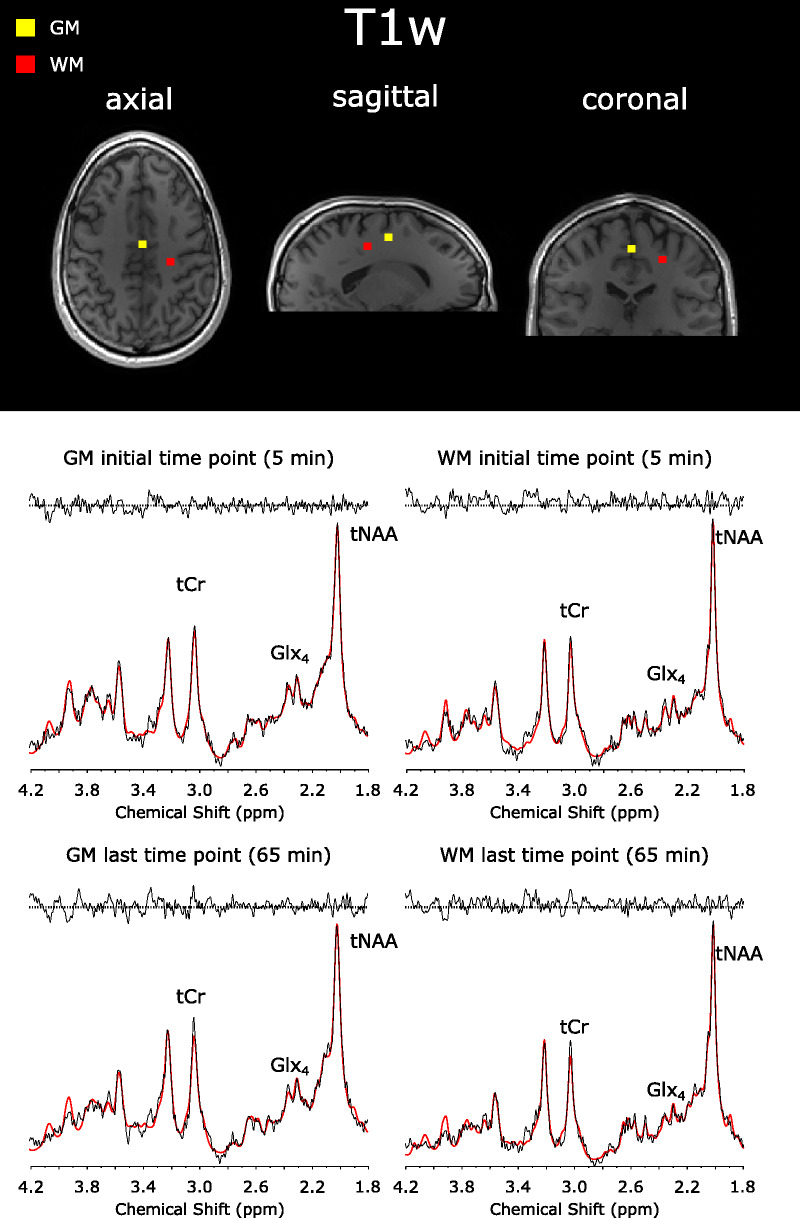
Sample spectra (black) and LCModel fit (red) from a single GM and WM voxel of 1 representative volunteer at the beginning (5 minutes) and the end (65 minutes) of the MRSI scan. Spectra were first-order corrected for illustration purposes. Relevant metabolites are total tCr, Glx, and tNAA. Spectral concentration fits of these particular voxels are shown in Supplemental Digital Content Table 1, http://links.lww.com/RLI/A798.

Sample spectra of single GM and WM voxels from the first and last time points of 1 representative subject are shown in Figure 3, with their respective metabolite concentrations and CRLBs (see Table 2).

**TABLE 2:** LCModel Spectral Fit Output of a Single Representative GM and WM Voxel for Selected Metabolites From the First (5 Minutes) and Last (60 Minutes) Time Points After d-Glucose Ingestion

Metabolite	GM Voxel		WM Voxel	
	Concentration, a.u. (CRLB, %)		Concentration, a.u. (CRLB, %)	
	5 Min	65 Min	5 Min	65 Min
tCr	1.07E4 (4%)	1.00E4 (5%)	7.47E3 (5%)	6.75E3 (4%)
tNAA	2.25E4 (3%)	2.25E4 (3%)	1.90E4 (3%)	2.03E4 (2%)
Glx4*	2.10E4 (7%)	1.53E4 (10%)	1.09E4 (10%)	9.00E3 (11%)
Glx23	2.68E4 (5%)	2.76E4 (5%)	1.36E4 (8%)	1.44E4 (6%)
Glu4*	1.44E4 (7%)	1.06E4 (10%)	7.77E3 (10%)	6.21E3 (12%)
Glu23	1.83E4 (7%)	1.82E4 (8%)	8.51E3 (11%)	6.60E3 (16%)
Gln4*	6.55E3 (12%)	4.63E3 (17%)	3.11E3 (18%)	2.80E3 (19%)
Gln23	8.46E3 (13%)	9.36E3 (13%)	5.11E3 (17%)	7.76E3 (12%)
Cr	7.13E3 (13%)	5.84E3 (17%)	5.58E3 (12%)	4.00E3 (15%)
PCr	3.60E3 (24%)	4.19E3 (24%)	1.89E3 (34%)	2.75E3 (22%)
NAA	2.12E4 (3%)	2.21E4 (3%)	1.69E4 (3%)	1.68E4 (3%)
NAAG	1.30E3 (30%)	4.15E2(111%)	2.17E3 (14%)	3.54E3 (9%)

Respective spectra and LCModel fits are shown in Figure 3. Data are for unlabeled metabolites unless otherwise indicated.

*Deuterium labeled.

GM indicates gray matter; WM, white matter; a.u., arbitrary units; tCr, total creatine; tNAA, total *N*-acetylaspartate; Glx, glutamate + glutamine; Glu, glutamate; Gln, glutamine; CRLB, Cramer-Rao lower bound; Cr, creatine; PCr, phosphocreatine; NAA, N-acetylaspartate; NAAG, N-acetylaspartylglutamic acid.

Voxel-wise linear fitting yielded moderate to strong (*r* < −0.5, *P* < 0.05) negative correlation between Glx_4_ signal intensity and time in 50% of GM and 44% of WM voxels from a 3D dataset of 1 representative participant (Supplementary Digital Content Fig. 4, http://links.lww.com/RLI/A798). Voxel-wise linear fitting was performed on data in arbitrary units to improve temporal stability. Slopes of the linear regression show a visible GM/WM contrast with 32% steeper slopes for Glx_4_ (*P* < 0.001) between GM and WM, representing a faster signal intensity decay, that is, higher metabolic activity.

**FIGURE 4:**
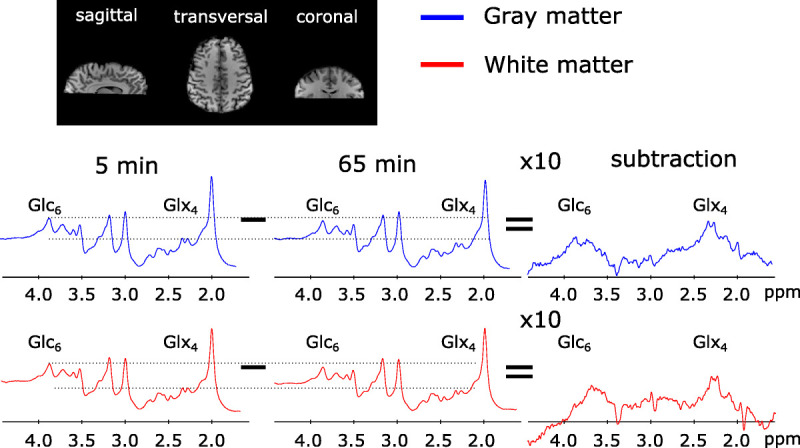
Averaged spectra over selected voxels in GM and WM 5 and 65 minutes after deuterium-labeled Glc administration visualizing a decrease in the Glx_4_ and Glc_6_ resonance, whereas other resonances were stable. Corresponding subtraction spectra showed an increase of the Glx_4_ and Glc_6_ resonance.

For illustration purposes, the subtraction spectra (first–last measurement) of averaged GM and WM voxels of 1 representative subject were calculated to visualize the signal drop of the Glx_4_ resonance 65 minutes after tracer administration as an increase of deuterium-labeled resonances only, similar to DMI (Fig. 4). Subtraction spectra showed Glx_4_ and Glc_6_ resonances. However, spectral fitting was performed voxel wise and without spectral subtraction.

More than 90% of GM and WM voxels featured CRLBs <20%, whereas overall, more than 60% of GM and WM voxels fulfilled the entire standardized quality criteria (ie, full width at half maximum <0.1 ppm, signal-to-noise ratio >15, CRLBs <20%).

## DISCUSSION

In this study, we have targeted the need for a fully noninvasive clinically applicable technique to map the dynamics of Glc metabolism in the human brain. We demonstrated the feasibility of our MR technique to image oxidative downstream Glc metabolism almost over the entire human cerebrum, using orally administered deuterium labeled Glc on a clinical routine 3T MR system.

The ultrashort echo time of the 3D FID-MRSI method minimizes J-evolution for metabolites such as Glx and improves the SNR compared with spin-echo approaches. High SNR and integrated real-time motion correction^15^ provided a high temporal stability reflected by a coefficient of variation of lower than 2% over the course of 60 minutes (14 time points) for regional averages of nonlabeled metabolites, that is, Glx_2 + 3_, tCr, and tNAA. This allowed for a reliable detection of 10% to 20% changes in signal amplitude for labeled metabolites (Glx_4_) due to deuterium enrichment even for voxel-wise analysis. However, regional averaging over multiple voxels improved the robustness of the results.

Excellent intrasubject repeatability was observed across scanners for static results at the beginning of the measurement, representing the repeatability of the method itself, without the influence of physiological variation (intrasubject coefficient of variation, 4% ± 4%). The decrease in Glx_4_ over time due to deuterium enrichment featured higher intrasubject variation for individual data points, whereas on average it was comparable. This could be explained presumably by physiological variation in Glc metabolism, as repeated measurements were performed up to 9 months apart.

Other approaches, for example, spectral fitting of subtraction spectra between time points, were not feasible as the coefficient of variation on voxel-wise level is too high to reliably detect a signal decrease of a few percentage (per time point) under low SNR conditions and missing reference peaks.

A proof that the decrease in Glx_4_ is not an artifact or systematic error of the method and indeed reflects physiological incorporation of deuterium into downstream metabolites has been performed in a previous study by assessing the test-retest repeatability and control measurements using regular Glc (dextrose), using a similar MR sequence (without motion correction) at 7T.^7^ In addition, results were compared with DMI data acquired from the same cohort of subjects and on the same MR scanner. The absolute decrease of Glx_4_ by −1.63 ± 0.3 mM in GM and −1.0 ± 0.3 mM in WM 60 minutes after labeled Glc administration is in good agreement with recent DMI literature.^5,26^ Therefore, no control measurements using unlabeled Glc were performed in this study. The relative decrease of Glx_4_ in our study is in good agreement with QELT literature.^6,8–10,27^

A 60% faster metabolic activity in GM compared with WM in our study is in line with differences reported in the rate of tricarboxylic acid cycle(68%) measured in ^13^C-studies^28,29^ but higher than [^18^F]FDG-PET literature values, which reported ~33% higher oxidative Glc consumption in GM than in WM.^30–32^ A visible decrease in the Glc_6_ resonance could be observed in Figure 4, but reliable fitting of the Glc resonances (labeled Glc_6_ and unlabeled Glc_1–5_) was not possible on voxel-wise level with comparable quality, presumably because of overlapping resonances in the region 3.5–4.0 ppm. For illustration purposes, metabolic map time courses without quality control thresholds are shown for all subjects in figure, Supplemental Digital Content 5, http://links.lww.com/RLI/A798. To calculate quantitative flux parameters of Glc metabolism, detection of the Glc uptake by detecting the decrease in Glc_6_ resonances needs to be improved, but the main aim of this study was a qualitative assessment of oxidative downstream metabolites, which directly reflects Glc utilization.

In contrast to a direct detection of labeled substrates using DMI (^2^H-MRS/MRSI), ^13^C-MRS, or [^18^F]FDG-PET the indirect ^1^H-based QELT approach allows for simultaneous detection of labeled and unlabeled metabolites to quantify an extended neurochemical profile and requires no additional hardware or radioactive tracers.^8–10^ In comparison with recent publications presenting QELT MRSI results in animals^9^ and humans^7,8,10^ at ultrahigh field(≥7T) research systems, our approach provides motion-corrected 3D metabolite maps with high temporal stability,^33^ which were acquired with high spatial and temporal resolution on widely available clinical 3T MR scanners. Further improvements in spatial and temporal stability can be anticipated considering the wide range of state-of-the-art undersampling or super-resolution techniques in MRSI.^34–36^

Similar to DMI, a separation of healthy oxidative and pathologic anaerobic metabolic pathways, via simultaneous detection of Glx and lactate, is theoretically feasible using ^1^H-MRSI. However, only 2% of Glc is anaerobically converted in the healthy human brain, and thus, we could not reliably quantify lactate concentrations in our study. Future applications could focus on pathologies involving anaerobic Glc utilization, for example, types of brain tumors, but as this introduces additional technical challenges, this would extend the scope of this study. A residual contamination from subcutaneous fat outside of the brain due to, for example, a suboptimal point spread function^18^ additionally limits the detection of lactate. Fat suppression,^37,38^ volume selective excitation using spin-echo approaches or higher spatial resolution,^39^ could improve lactate detection in future studies involving pathologies with high lactate production,^40^ but we will further investigate the feasibility of lactate detection using our ^1^H FID MRSI sequence via simulations and experimental validation in the future.

Although our study shows only results for investigating Glc metabolism, deuterium labeling is not limited to Glc. A range of metabolites can be deuterium-labeled, for example, choline, acetate^41^ to study different metabolic pathways and pathologies—some of them even simultaneously using multiple tracers, for example, in tumors.^42^ This adds additional flexibility for designing clinical research studies. Deuterium-labeled Glc ([6,6′]-^2^H-Glc) is a safe and stable tracer and the price of the administered dose was under $1000 for each subject. However, this price is expected to drop upon mass production and the approval of cheaper approaches to synthesize, for example, deuterated Glc.^43,44^

Because of missing medical personnel, we were only able to sample blood Glc for 5 of 7 healthy participants for the initial measurement bout.

Oral administration was preferred over intravenous injection in favor of participant comfort. Compared with intravenous injection, an expected delayed response and slower increase in blood Glc isotopic enrichment after oral Glc uptake, together with a non-steady-state of blood Glc and relatively short measurement time limits possible calculation of quantitative flux rates,^45^ which was not the aim of this study. Intravenous tracer injection would allow additional blood sampling to estimate isotopic enrichment time courses as an input function to overcome those challenges in future studies.^27^ A previous study^7^ using DMI showed that the increase in deuterium-labeled Glx features an approximately linear behavior in the first 60 minutes after oral administration; therefore, our study used linear fitting as an approximation to estimate the dynamics of Glx, but this approach does not reflect real quantitative turnover rates. Other models, such as exponential fitting, has been applied previously but require longer scan times (eg, 2–3 hours) and the assessment of a true baseline. However, whether accurately quantifying flux rates via a lengthy acquisition provides sufficient added diagnostic value in patients remains to be shown.

In general, the presented measurement time of 60 minutes could still be considered too long for clinical application. Splitting the experiment into a short pre– (baseline) and post–Glc administration measurement after a defined pause would significantly reduce patient scan time.

We do not expect that subject repositioning would severely affect the quality and reliability of the data based on recent test-retest results at 7T^46,47^ and extrapolating expected quality changes from 7T to 3T based on comparison data.^48,49^

This study not only demonstrates the feasibility of dynamic 3D ^1^H-MRSI to noninvasively image Glc downstream metabolism in the human brain using deuterium-labeled Glc at widely available clinical 3T MR scanners but also suggests the possibility to map several other deuterated downstream metabolites of clinical interest.

## Supplementary Material

## References

[1] Koppenol WH Bounds PL Dang CV. Otto Warburg's contributions to current concepts of cancer metabolism. *Nat Rev Cancer*. 2011;11:325–337. 21508971 10.1038/nrc3038

[2] Norat P Soldozy S Sokolowski JD, . Mitochondrial dysfunction in neurological disorders: exploring mitochondrial transplantation. *NPJ Regen Med*. 2020;5:22. 33298971 10.1038/s41536-020-00107-x PMC7683736

[3] Manji H Kato T Di Prospero NA, . Impaired mitochondrial function in psychiatric disorders. *Nat Rev Neurosci*. 2012;13:293–307. 22510887 10.1038/nrn3229

[4] Kumar M Nanga RPR Verma G, . Emerging MR imaging and spectroscopic methods to study brain tumor metabolism. *Front Neurol*. 2022;13:789355. 35370872 10.3389/fneur.2022.789355 PMC8967433

[5] De Feyter HM Behar KL Corbin ZA, . Deuterium metabolic imaging (DMI) for MRI-based 3D mapping of metabolism in vivo. *Sci Adv*. 2018;4:eaat7314. 30140744 10.1126/sciadv.aat7314 PMC6105304

[6] Ruhm L Avdievich N Ziegs T, . Deuterium metabolic imaging in the human brain at 9.4 tesla with high spatial and temporal resolution. *Neuroimage*. 2021;244:118639. 34637905 10.1016/j.neuroimage.2021.118639 PMC8591372

[7] Bednarik P Goranovic D Svatkova A, . Deuterium labeling enables non-invasive 3D proton MR imaging of glucose and neurotransmitter metabolism in the human brain at 7 T. *Nat Biomed Eng*. 2022.

[8] Cember ATJ Wilson NE Rich LJ, . Integrating (1)H MRS and deuterium labeled glucose for mapping the dynamics of neural metabolism in humans. *Neuroimage*. 2022;251:118977. 35143973 10.1016/j.neuroimage.2022.118977 PMC9166154

[9] Rich LJ Bagga P Wilson NE, . ^1^H magnetic resonance spectroscopy of ^2^H-to-^1^H exchange quantifies the dynamics of cellular metabolism in vivo. *Nat Biomed Eng*. 2020;4:335–342. 31988460 10.1038/s41551-019-0499-8 PMC7071956

[10] Ruhm L Ziegs T Wright AM, . Dynamic observation of ^2^H labeled compounds in the human brain with ^1^H versus ^2^H magnetic resonance spectroscopy at 9.4 T. *bioRxiv*. 2022. 2022.01.24.477582.

[11] Boumezbeur F Besret L Valette J, . NMR measurement of brain oxidative metabolism in monkeys using 13C-labeled glucose without a 13C radiofrequency channel. *Magn Reson Med*. 2004;52:33–40. 15236364 10.1002/mrm.20129

[12] Kaggie JD Khan AS Matys T, . Deuterium metabolic imaging and hyperpolarized (13)C-MRI of the normal human brain at clinical field strength reveals differential cerebral metabolism. *Neuroimage*. 2022;257:119284. 35533826 10.1016/j.neuroimage.2022.119284

[13] Bogner W Hess AT Gagoski B, . Real-time motion- and B0-correction for LASER-localized spiral-accelerated 3D-MRSI of the brain at 3T. *Neuroimage*. 2014;88:22–31. 24201013 10.1016/j.neuroimage.2013.09.034 PMC4010560

[14] Hingerl L Strasser B Moser P, . Clinical high-resolution 3D-MR spectroscopic imaging of the human brain at 7T. *Invest Radiol*. 2020;55:239–248. 31855587 10.1097/RLI.0000000000000626

[15] Moser P Eckstein K Hingerl L, . Intra-session and inter-subject variability of 3D-FID-MRSI using single-echo volumetric EPI navigators at 3T. *Magn Reson Med*. 2020;83:1920–1929. 31721294 10.1002/mrm.28076 PMC7065144

[16] Lin A Andronesi O Bogner W, . Minimum reporting standards for in vivo magnetic resonance spectroscopy (MRSinMRS): experts' consensus recommendations. *NMR Biomed*. 2021;34:e4484. 33559967 10.1002/nbm.4484 PMC8647919

[17] Ogg RJ Kingsley PB Taylor JS. WET, a T1- and B1-insensitive water-suppression method for in vivo localized 1H NMR spectroscopy. *J Magn Reson B*. 1994;104:1–10. 8025810 10.1006/jmrb.1994.1048

[18] Hingerl L Bogner W Moser P, . Density-weighted concentric circle trajectories for high resolution brain magnetic resonance spectroscopic imaging at 7T. *Magn Reson Med*. 2018;79:2874–2885. 29106742 10.1002/mrm.26987 PMC5873433

[19] Bilgic B Chatnuntawech I Fan AP, . Fast image reconstruction with L2-regularization. *J Magn Reson Imaging*. 2014;40:181–191. 24395184 10.1002/jmri.24365 PMC4106040

[20] Strasser B Chmelik M Robinson SD, . Coil combination of multichannel MRSI data at 7T: MUSICAL. *NMR Biomed*. 2013;26:1796–1805. 24038331 10.1002/nbm.3019 PMC3912904

[21] Povazan M Hangel G Strasser B, . Mapping of brain macromolecules and their use for spectral processing of (1)H-MRSI data with an ultra-short acquisition delay at 7T. *Neuroimage*. 2015;121:126–135. 26210813 10.1016/j.neuroimage.2015.07.042

[22] Choi C Coupland NJ Bhardwaj PP, . T2 measurement and quantification of glutamate in human brain in vivo. *Magn Reson Med*. 2006;56:971–977. 17029225 10.1002/mrm.21055

[23] Gasparovic C Song T Devier D, . Use of tissue water as a concentration reference for proton spectroscopic imaging. *Magn Reson Med*. 2006;55:1219–1226. 16688703 10.1002/mrm.20901

[24] Mlynarik V Gruber S Moser E. Proton T (1) and T (2) relaxation times of human brain metabolites at 3 tesla. *NMR Biomed*. 2001;14:325–331. 11477653 10.1002/nbm.713

[25] Jenkinson M Beckmann CF Behrens TE, . FSL. *Neuroimage*. 2012;62:782–790. 21979382 10.1016/j.neuroimage.2011.09.015

[26] Seres Roig E De Feyter HM Nixon TW, . Deuterium metabolic imaging of the human brain in vivo at 7T. *Magn Reson Med*. 2023;89:29–39. 36063499 10.1002/mrm.29439 PMC9756916

[27] Moreno A Bluml S Hwang JH, . Alternative 1-(13)C glucose infusion protocols for clinical (13)C MRS examinations of the brain. *Magn Reson Med*. 2001;46:39–48. 11443709 10.1002/mrm.1158

[28] Pan JW Stein DT Telang F, . Spectroscopic imaging of glutamate C4 turnover in human brain. *Magn Reson Med*. 2000;44:673–679. 11064400 10.1002/1522-2594(200011)44:5<673::aid-mrm3>3.0.co;2-l

[29] Shulman RG Rothman DL Behar KL, . Energetic basis of brain activity: implications for neuroimaging. *Trends Neurosci*. 2004;27:489–495. 15271497 10.1016/j.tins.2004.06.005

[30] Hyder F Fulbright RK Shulman RG, . Glutamatergic function in the resting awake human brain is supported by uniformly high oxidative energy. *J Cereb Blood Flow Metab*. 2013;33:339–347. 23299240 10.1038/jcbfm.2012.207 PMC3587823

[31] Hyder F Rothman DL. Quantitative fMRI and oxidative neuroenergetics. *Neuroimage*. 2012;62:985–994. 22542993 10.1016/j.neuroimage.2012.04.027 PMC3389300

[32] Yu Y Herman P Rothman DL, . Evaluating the gray and white matter energy budgets of human brain function. *J Cereb Blood Flow Metab*. 2018;38:1339–1353. 28589753 10.1177/0271678X17708691 PMC6092772

[33] Andronesi OC Bhattacharyya PK Bogner W, . Motion correction methods for MRS: experts' consensus recommendations. *NMR Biomed*. 2021;34:e4364. 33089547 10.1002/nbm.4364 PMC7855523

[34] Bogner W Otazo R Henning A. Accelerated MR spectroscopic imaging—a review of current and emerging techniques. *NMR Biomed*. 2021;34:e4314. 32399974 10.1002/nbm.4314 PMC8244067

[35] Iqbal Z Nguyen D Hangel G, . Super-resolution (1)H magnetic resonance spectroscopic imaging utilizing deep learning. *Front Oncol*. 2019;9:1010. 31649879 10.3389/fonc.2019.01010 PMC6794570

[36] Hangel G Jain S Springer E, . High-resolution metabolic mapping of gliomas via patch-based super-resolution magnetic resonance spectroscopic imaging at 7 T. *Neuroimage*. 2019;191:587–595. 30772399 10.1016/j.neuroimage.2019.02.023 PMC7220803

[37] Tkac I Deelchand D Dreher W, . Water and lipid suppression techniques for advanced (1) H MRS and MRSI of the human brain: experts' consensus recommendations. *NMR Biomed*. 2021;34:e4459. 33327042 10.1002/nbm.4459 PMC8569948

[38] Hangel G Strasser B Povazan M, . Lipid suppression via double inversion recovery with symmetric frequency sweep for robust 2D-GRAPPA-accelerated MRSI of the brain at 7T. *NMR Biomed*. 2015;28:1413–1425. 26370781 10.1002/nbm.3386 PMC4973691

[39] Hangel G Strasser B Povazan M, . Ultra-high resolution brain metabolite mapping at 7 T by short-TR Hadamard-encoded FID-MRSI. *Neuroimage*. 2018;168:199–210. 27825954 10.1016/j.neuroimage.2016.10.043

[40] Maudsley AA Andronesi OC Barker PB, . Advanced magnetic resonance spectroscopic neuroimaging: experts' consensus recommendations. *NMR Biomed*. 2021;34:e4309. 32350978 10.1002/nbm.4309 PMC7606742

[41] Polvoy I Qin H Flavell RR, . Deuterium metabolic imaging—rediscovery of a spectroscopic tool. *Metabolites*. 2021;11:570. 34564385 10.3390/metabo11090570 PMC8470013

[42] Veltien A van Asten J Ravichandran N, . Simultaneous recording of the uptake and conversion of glucose and choline in tumors by deuterium metabolic imaging. *Cancers (Basel)*. 2021;13:4034. 34439188 10.3390/cancers13164034 PMC8394025

[43] Fujiwara Y Iwata H Sawama Y, . Method for regio-, chemo- and stereoselective deuterium labeling of sugars based on ruthenium-catalyzed C-H bond activation. *Chem Commun (Camb)*. 2010;46:4977–4979. 20544123 10.1039/c0cc01197e

[44] Li W Rabeah J Bourriquen F, . Scalable and selective deuteration of (hetero)arenes. *Nat Chem*. 2022;14:334–341. 35027706 10.1038/s41557-021-00846-4 PMC8898765

[45] Kreis F Wright AJ Hesse F, . Measuring tumor glycolytic flux in vivo by using fast deuterium MRI. *Radiology*. 2020;294:289–296. 31821119 10.1148/radiol.2019191242

[46] Hangel G Spurny-Dworak B Lazen P, . Inter-subject stability and regional concentration estimates of 3D-FID-MRSI in the human brain at 7T. *NMR Biomed*. 2021;34:e4596. 34382280 10.1002/nbm.4596 PMC11475238

[47] Heckova E Považan M Strasser B, . Effects of different macromolecular models on reproducibility of FID-MRSI at 7T. *Magn Reson Med*. 2020;83:12–21. 31393037 10.1002/mrm.27922 PMC6851974

[48] Gruber S Heckova E Strasser B, . Mapping an extended neurochemical profile at 3 and 7T using accelerated high-resolution proton magnetic resonance spectroscopic imaging. *Invest Radiol*. 2017;52:631–639. 28459799 10.1097/RLI.0000000000000379

[49] Motyka S Moser P Hingerl L, . The influence of spatial resolution on the spectral quality and quantification accuracy of whole-brain MRSI at 1.5 T, 3T, 7T, and 9.4 T. *Magn Reson Med*. 2019;82:551–565. 30932248 10.1002/mrm.27746 PMC6563461

