# Supplementary material for: Noninvasive 3-Dimensional 1H-Magnetic Resonance Spectroscopic Imaging of Human Brain Glucose and Neurotransmitter Metabolism Using Deuterium Labeling at 3T: Feasibility and Interscanner Reproducibility
Source: Invest Radiol. 2023 Feb 4;58(6):431–7. doi: 10.1097/RLI.0000000000000953 (PMC10184811; doi:10.1097/RLI.0000000000000953)
Supplement: Supplementary file 1 [file ir-58-431-s001.pdf]

Minimum Reporting Standards in MR Spectroscopy checklist (according to Lin et al. NMR Biomed 2021)	
**1. Hardware**	
**a. Field strength [T]**	3
**b. Manufacturer**	Siemens
**c. Model (software version if available)**	Prisma Fit
**d. RF coils: nuclei (transmit/ receive), number of channels, type, body part**	1H RX 64 channels, head, Siemens 1H TX: body coil
**e. Additional hardware**	N/A
**2. Acquisition**	
**a. Pulse sequence**	FID-CRT MR spectroscopic imaging
**b. Volume of Interest (VOI) locations**	The excited 55 mm-thick slab was centered around the posterior cingulate region.
**c. Nominal VOI size [cm³, mm³]**	200×200×55 mm³
**d. Repetition Time (TR), Echo Time (TE) [ms, s]**	TR=950 ms / 0.8 ms acquisition delay
**e. Total number of Excitations or acquisitions per spectrum**	1 average
**In time series for kinetic studies**	N/A
**i. Number of Averaged spectra (NA) per time-point**	N/A
**ii. Averaging method (e.g. block-wise or moving average)**	N/A
**iii. Total number of spectra (acquired / in time-series)**	N/A
**f. Additional sequence parameters (spectral width in Hz, number of spectral points, frequency offsets); If STEAM: Mixing Time TM; If MRSI: 2D or 3D, FOV in all directions, matrix size, acceleration factors**	Bandwidth: 1325 Hz, 588 spectral points, MRSI: 3D, FOV 200×200x130 mm², Matrix size: 32x32x21
**g. Water Suppression Method**	WET
**h. Shimming Method, reference peak, and thresholds for "acceptance of shim" chosen**	Standard shim + manual adjustment, water peak < 30 Hz
**i. Triggering or motion correction method (respiratory, peripheral, cardiac triggering, incl. device used and delays)**	N/A
**3. Data analysis methods and outputs**	
**a. Analysis software**	LCModel 6.3-1
**b. Processing steps deviating from quoted reference or product**	N/A
**c. Output measure (e.g. absolute concentration, institutional units, ratio)**	institutional units, ratio
**d. Quantification references and assumptions, fitting model assumptions**	Simulated in NMRScope-B, macromolecular background
**4. Data Quality**	
**a. Reported variables (SNR, Linewidth (with reference peaks))**	SNR was calculated using the pseudoreplica method, and linewidth as FWHM of the NAA fit
**b. Data exclusion criteria**	CRLBs >20% for tNAA, tCr and Glu+Gln (Glx)
**c. Quality measures of postprocessing Model fitting (e.g. CRLB, goodness of fit, SD of residual)**	CRLB
**d. Sample Spectrum**	See Supplemental Digital Content Figure 2

Supplemental Digital Content Table 1: Minimum Reporting Standards for in vivo MR Spectroscopy

Note. – CRLB = Cramér-Rao lower bounds; FID = free induction decay; FOV = field of view; FWHM = full-width-at-half-maximum; Glu = Glutamate; Gln = Glutamine; tNAA = total N-acetylaspartate; SNR = signal-to-noise ratio; tCr = total creatine; VOI = volume of interest.

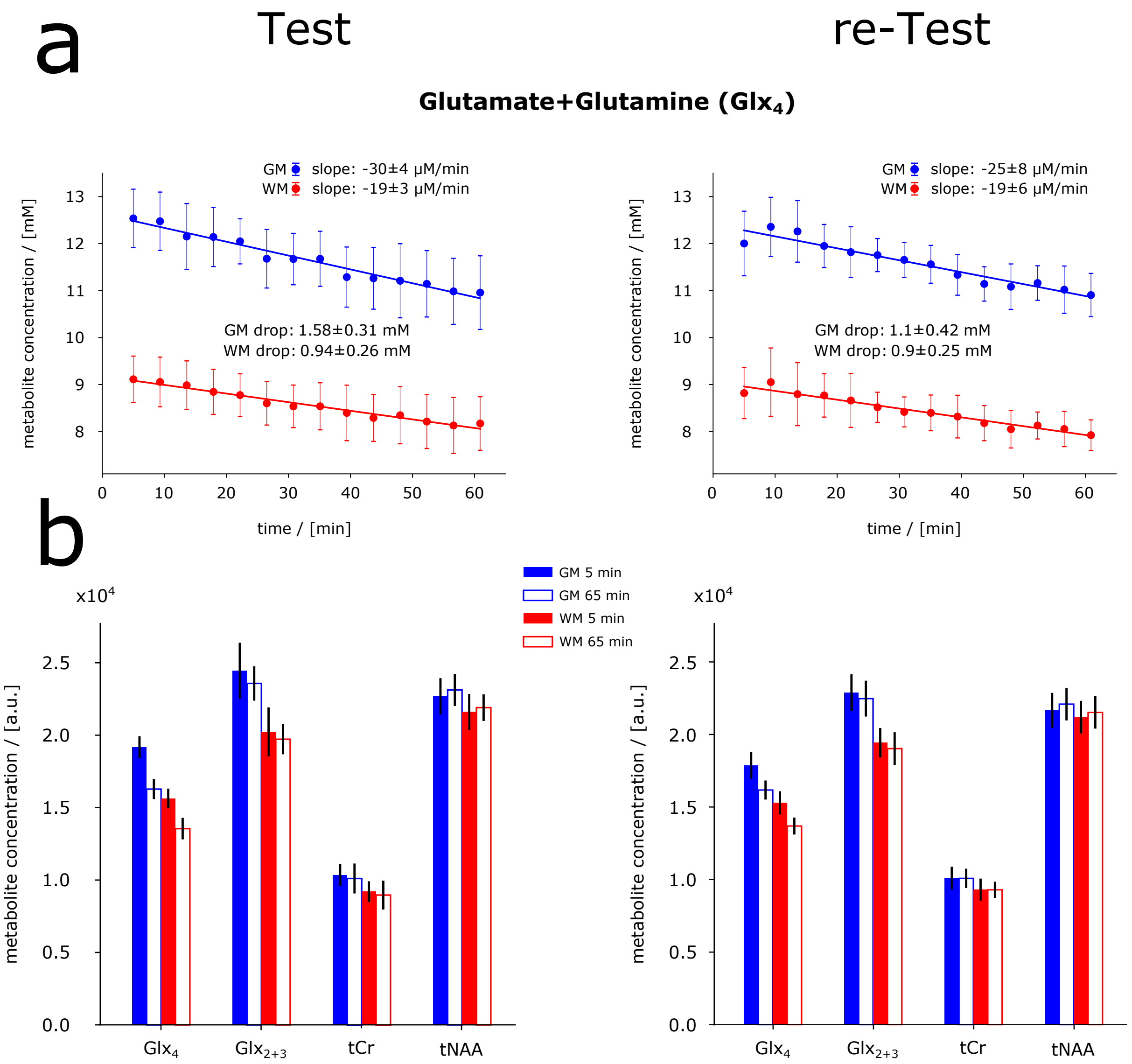

Supplementary Digital Content Figure 1:

Test and Re-test comparison between six out of seven original participants. Time courses and linear regression analysis of combined glutamate+glutamine (Glx) concentratios (in mM) regionally averaged over gray and white matter voxels and over all participants (a). Results suggest an overall good repeatability of the concentration estimates and rates of Glx decrease were not significantly different. Comparison between test and retest measurement for non-labeled metabolites concentrations (arbitrary units a.u.): Glx_{2+3}, total creatine (tCr) and total N-acetylaspartate (tNAA) (b).

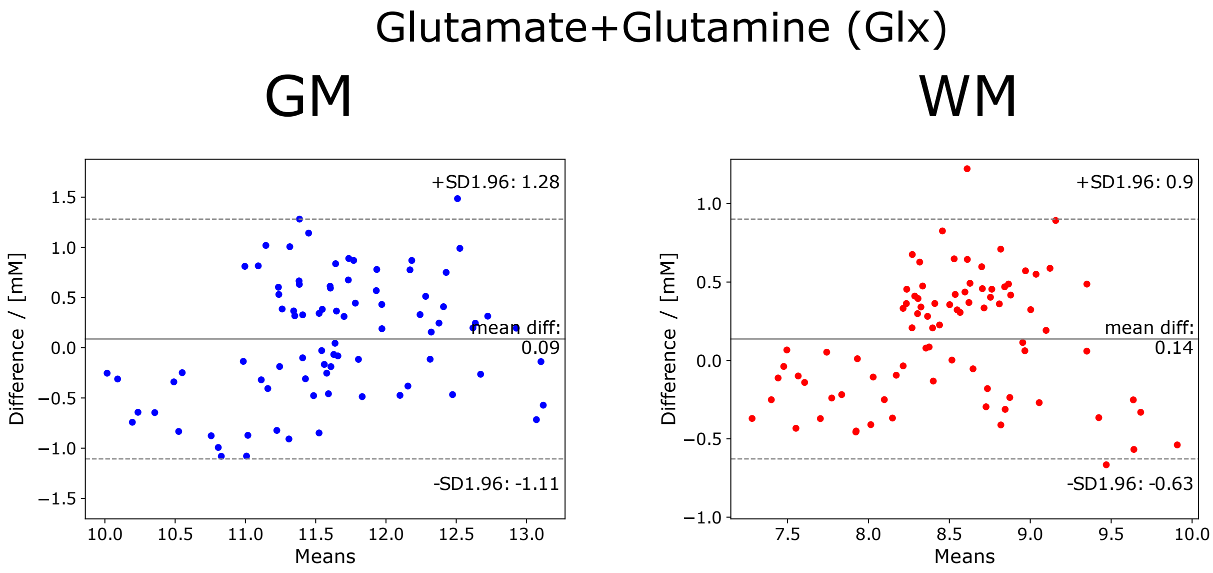

Supplementary Digital Content Figure 2: Bland-Altman comparison for test and re-test measurement bouts of combined Glutamate+Glutamine (Glx) concentrations in mM regionally averaged over gray and white matter and over all re-measured participants (n=6).

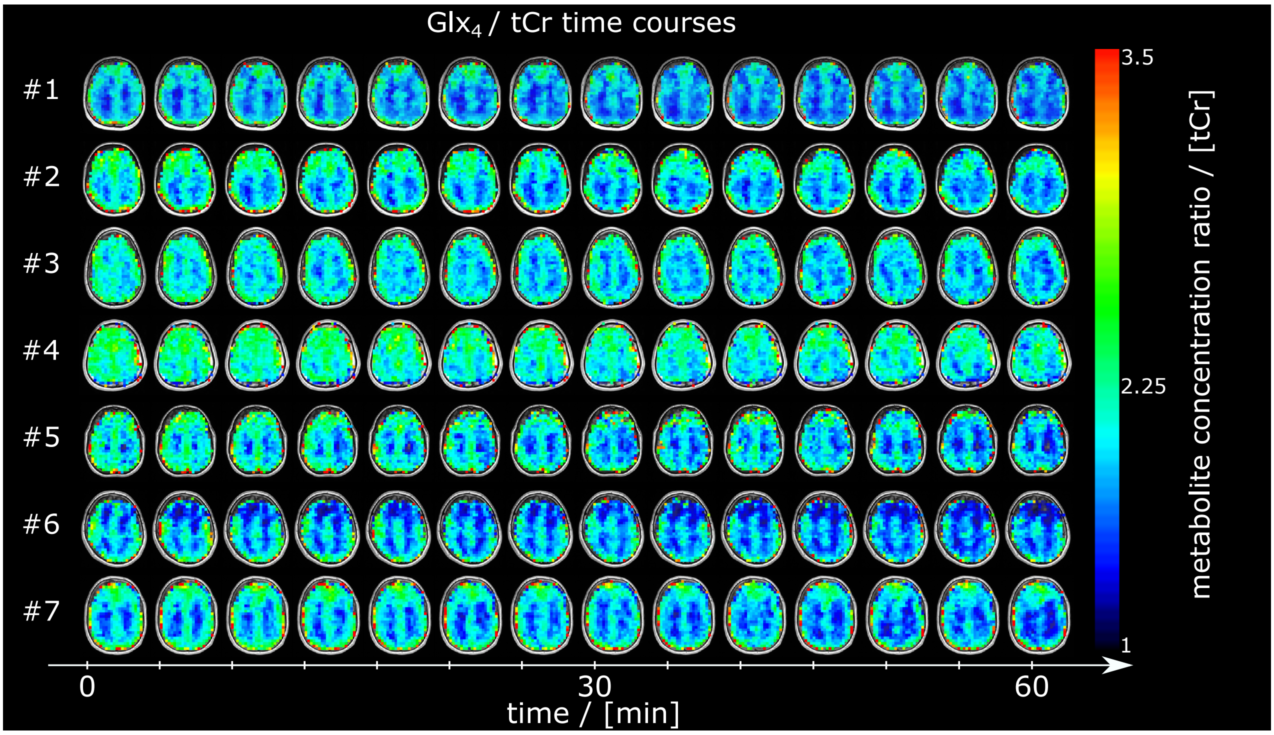

Supplemental Digital Content Figure 3: Time courses of axial glutamate+glutamine (Glx$_4$) ratio maps referenced to total creatine from all subjects over the entire measurement visualizing the image intensity decrease over time due to deuterium labeling.

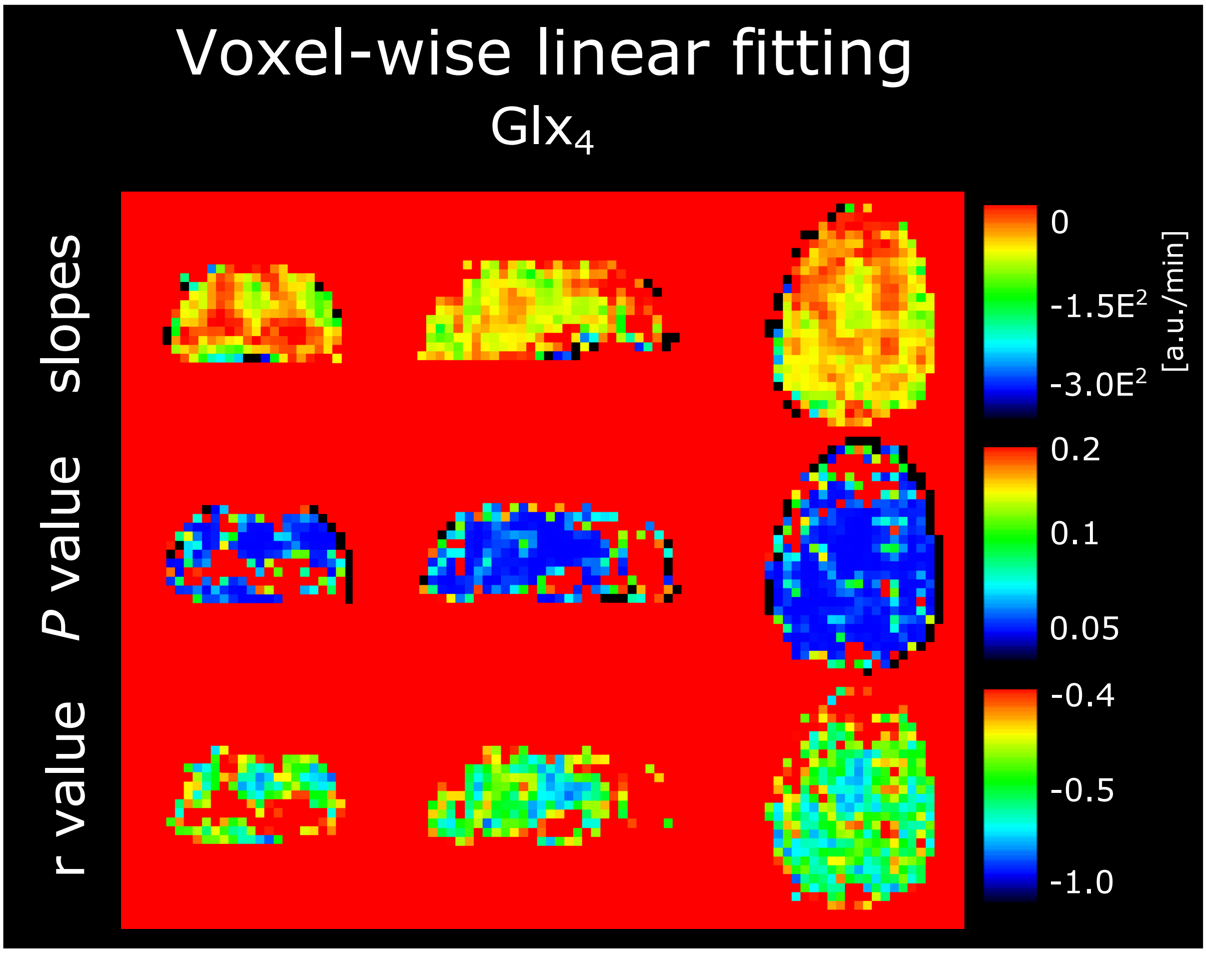

Supplementary Digital Content Figure 4: Voxel-wise linear fitting between time and deuterium labeled metabolites, i.e., glutamate+glutamine (Glx_4) shown from one representative participant featuring slopes, *P* values r values (top,middle, bottom row respectively) for each voxel separately of the whole 3D volume. The slope map shows a contrast between GM and WM with 32% steeper slopes for Glx_4 ($p<0.001$) in GM versus WM. Linear fitting was statistically significant ($p < 0.05$) with correlation ($r < -0.5$) in 50% and 44% of GM and WM voxels, respectively.

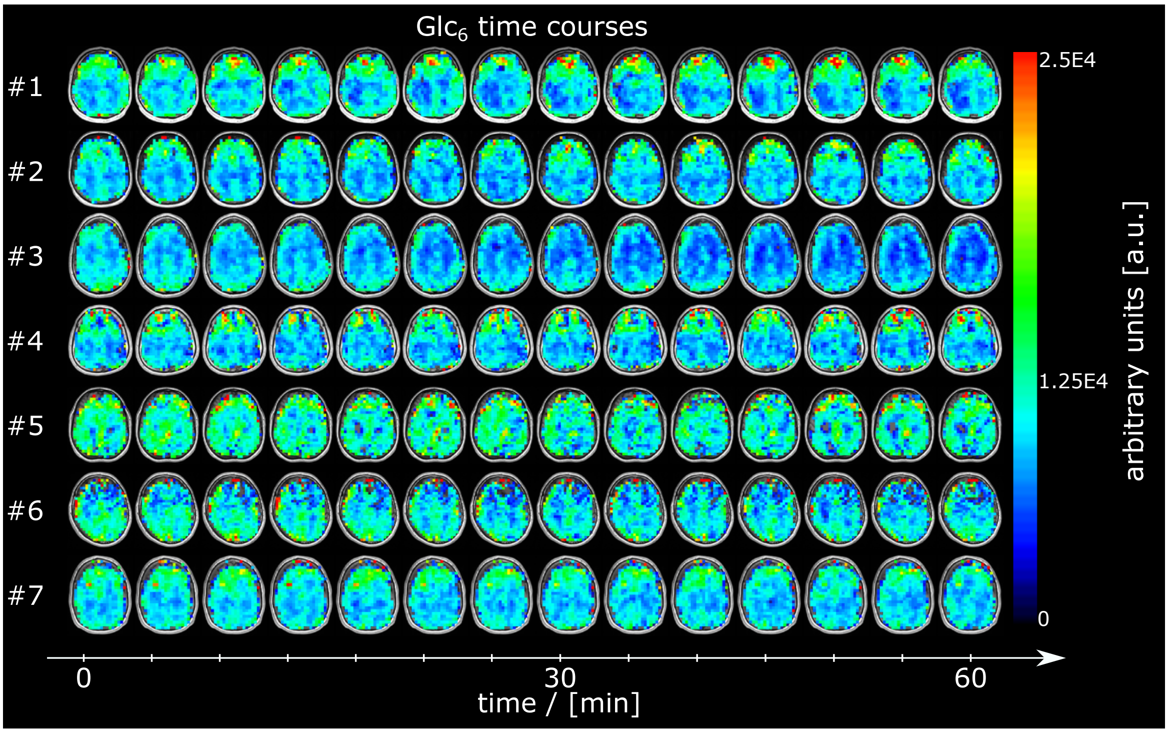

Supplemental Digital Content Figure 5: Time courses of axial glucose (Glc_6) maps (in arbitrary units a.u.) from all subjects over the entire measurement visualizing the image intensity decrease over time due to deuterium labeling. No quality criteria threshold was applied for Glc_6 data.
